# Aesthetic Reconstruction of Onco-surgical Mandibular Defects Using Free Fibular Flap with and without CAD/CAM Customized Osteotomy Guide: A Randomized Controlled Clinical Trial

**DOI:** 10.1186/s12885-022-10322-y

**Published:** 2022-12-02

**Authors:** Mohammed Esmail Al-Sabahi, Omer Mohammed Jamali, Mostafa Ibrahim Shindy, Basma Gamal Moussa, Ayman Abdel-Wahab Amin, Mohamed Hamdallah Zedan

**Affiliations:** 1 Department of Oral and Maxillofacial Surgery, Faculty of Dentistry, Cairo University, Cairo, Egypt; 2 Present Address: Department of Oral and Maxillofacial Surgery, Faculty of Dentistry, Ibb University, Ibb, Yemen; 3 Department of Oral and Maxillofacial Surgery, Faculty of Dentistry, Hodeidah University, Hodeidah, Yemen; 4 Department of Surgical Oncology, Division of Head and Neck Surgery, National Cancer Institute, Cairo University, Cairo, Egypt

**Keywords:** Aesthetic reconstruction, CAD/CAM, Customized osteotomy/cutting guide, Free fibula flap, Head and neck cancer, Mandibular defects, Model-based reconstruction, Virtual planning

## Abstract

**Background:**

Reconstruction of mandibular defects following ablative surgery remains a challenge even for experienced surgeons. Virtual planning and guided surgery, including computer-aided design/computer-aided manufacturing (CAD/CAM), afford optimized ways by which to plan complex surgery. This study aimed to evaluate and compare aesthetic outcome and surgical efficiency of free fibular flap (FFF) with and without CAD/CAM customized osteotomy guide (COG) for reconstruction of onco-surgical mandibular defects.

**Methods:**

Twenty-two patients indicated for segmental mandibulectomy were randomly assigned to either CAD/CAM with COG group or that without COG- Model based reconstruction (MB group) at a 1:1 ratio. Aesthetic outcomes were evaluated by means of morphometric assessment and comparison for each differential area (DAr) and angle (DAn) in the affected side to the contralateral side of the mandible using computerized digital imaging analysis (CDIA) based on the post-operative 3D CT-scan. Subjective evaluation was performed using the Visual Analogue Scale (VAS) and Patient’s Satisfaction Score (PSS). Surgical efficiency was a secondary outcome and evaluated as total operative time and ischemia time.

**Results:**

The mean sagittal DAr was significantly lower in the COG group (277.28 ± 127.05 vs. 398.67 ± 139.10 mm^2^, *P* = 0.045). Although there was an improvement in the axial DAr (147.61 ± 55.42 vs. 183.68 ± 72.85 mm^2^), the difference was not statistically significant (*P* = 0.206). The mean differences (Δ) in both sagittal and coronal DAn were significantly lower in the COG group than in the MB group (6.11 ± 3.46 and 1.77 ± 1.12° vs. 9.53 ± 4.17 and 3.44 ± 2.34°), respectively. There were no statistically significant differences in the axial DAn between the two groups (*P* = 0.386). The PSS was significantly higher in the COG group, reflecting better aesthetic satisfaction than in the MB group (*P* = 0.041). The total operation and ischemia time were significantly shorter in favor of the COG group with a mean of (562.91 ± 51.22, 97.55 ± 16.80 min vs. 663.55 ± 53.43, 172.45 ± 21.87 min), respectively.

**Conclusion:**

The CAD/CAM with COG is more reliable and highly valuable in enhancing aesthetic outcomes and surgical efficiency of mandibular reconstruction by FFF compared to that without COG (MB reconstruction).

**Trial registration:**

This trial was registered at ClinicalTrials.gov. Registration number: NCT03757273. Registration date: 28/11/2018.

## Background

Maxillofacial defects resulting from surgical resection of neoplasms can be associated with devastating functional and aesthetic deficits. Taking into consideration the mandible’s importance as a major determinant in facial aesthetics and function; disruption of mandibular integrity following onco-surgical resection can create extensive composite defects, leading to functional and aesthetic impairments [1–3].

Facial deformities affecting the lower facial contour can have major consequences. Therefore, the reconstruction process should be highly considered to limit further repercussions and thus avoid concurrent impairments [4]. Reconstruction of such mandibulectomy defects continues to be extremely demanding and challenging for reconstructive surgeons intending both functional and aesthetic restoration with the least surgical morbidity [1].

Various options and modalities for reconstruction have been reported. Currently, the microvascular fibular flap has been established as the gold standard for optimal onco-mandibular defects reconstruction [5]. The main goals of mandibular reconstruction are to obtain a structural replacement with restoration of facial symmetry and functions. The face is an individual’s interface with society, and the aesthetic result should be the best possible. Therefore, re-establishing the form of the lower third of the face is extremely important for facial aesthetics, as the mandible forms its’ bony foundation [6–9]. Before the advent of computer-assisted surgery (CAS), the traditional/conventional method was based on a freehand approach for segmental osteotomies. Conforming flap to defect was a complex and crucial task that involved a tedious learning curve, and results varied greatly between surgeons according to experience and technical skills. In recent years, Virtual surgical planning (VSP) including, Computer-Aided Design and Manufacturing (CAD-CAM), has significantly revolutionized and changed the way of bony reconstruction [10, 11].

Aesthetic outcomes are one of the main goals of mandibular reconstruction. However, standard criteria for measuring these outcomes are currently insufficient [12]*.* Earlier forms of evaluation were based on pantomography to evaluate mandibular symmetry [5, 13, 14]. Current methods of assessment are based on 3D imaging analysis. Nevertheless, standardization regarding the evaluation process is still a concern. In the literature, most studies have focused on the accuracy of the transfer from pre-operative planning to post-operative implementation, with few making a comparison between the VSP cases and a control group [11].

Furthermore, all of those comparative studies have used conventional technique as a control group or still consider accuracy rather than symmetry, thus aesthetics. There are currently no published randomized clinical studies that have compared the aesthetic outcome of the FFF using VSP with that of the MB technique. Therefore, the aim of this study was to evaluate and compare the aesthetic outcomes and surgical efficiency of mandibular reconstruction using FFF with CAD/CAM COG to that without COG (MB reconstruction) after mandibulectomy in patients with mandibular tumors.

## Patients and methods

### Study Setting

This study was conducted in the National Cancer Institute (NCI) and Faculty of Dentistry, Cairo University, Egypt, from November 2018 to December 2021. A total of 22 patients with primary mandibular tumors and indicated for segmental resection were recruited for randomization, regardless of age, sex, and ethnicity. Patients with poor oncological prognosis, poor performance status, or those with relative or absolute vascular contraindications for FFF were excluded. Patients indicated for double free-flap reconstruction or those who just required marginal resection were also excluded from this study. Patients were evaluated preoperatively by clinical history, physical examination, CT scans for the craniofacial skeleton and lower extremities (bilateral fibula angiographic CT scan), and biopsy from the primary lesion. This study was approved by the Research Ethical Committee at the Faculty of Dentistry, Cairo University (identifier 24/12/18) and was conducted in accordance with the Helsinki Declaration. Written informed consent was obtained from all patients. The trial was registered at ClinicalTrials.gov with identifier NCT03757273. Registration date: 28/11/2018. The 2010 CONSORT statement and guidelines for reporting parallel-group randomized trials were applied in the reporting of this study.

### Trial Design

This was a prospective, randomized, parallel, controlled clinical study. The participants were screened by two investigators (M.E.A. and M.H.Z.). The randomization sequence was created on www.randomization.com with a 1:1 allocation using random block sizes of 2 and 4. Allocation concealment was implemented with sequentially numbered, opaque sealed envelopes (SNOSE). After preoperative evaluation, the envelope with the previously generated sequence was opened by a clinician not involved in the study, and the patient was randomly assigned to one of two groups; Group I comprised 11 patients whose mandibular reconstruction was performed using FFF with the COG technique (COG group). Group II comprised 11 patients whose mandibular reconstruction was performed using FFF with the MB technique (MB group). Because the two interventions used in this trial were easily recognized by the investigators, only the outcome assessor was blinded.

### Treatment

#### VSP and Design of Guiding Templates

**Fig. 1:**
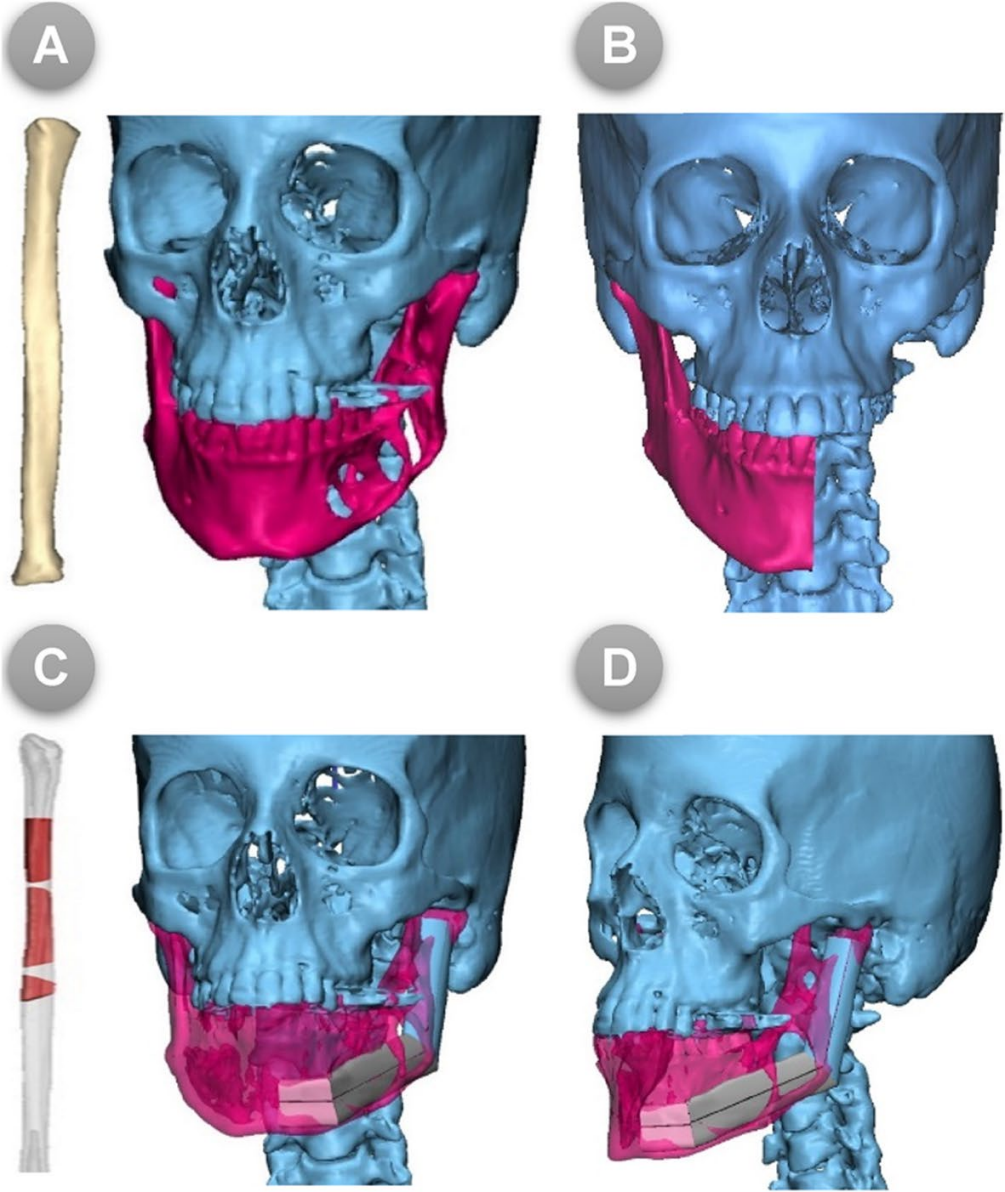
(**A**) 3D virtual models of the craniofacial skeleton, (**B**) Virtual planned mandibular resection in frontal views, (**C**) 3D virtual models of the bony fibula and mandible with planned fibular osteotomies, (**D**) 3D reconstructed fibula was superimposed on the mandibular defect in lateral view

The acquired CT images with fin cuts (< 1 mm) in DICOM format were imported into the Synthes ProPlan CMF™ software, V. 3.0 (Materialise® NV, Technologielaan 15, 3001 Leuven, Belgium. https://www.materialise.com/en/medical/software/proplan-cmf) to be processed and transformed for the creation of three-dimensional (3D) virtual models of the craniofacial skeleton and the bony fibula **(**Fig. 1A**)**. In the interactive surgical planning, the process of virtual mandibular resection and fibular osteotomies were clearly mapped; this was fulfilled via the designer-surgeon communication and work to confirm the osteotomy lines together.

**Fig. 2:**
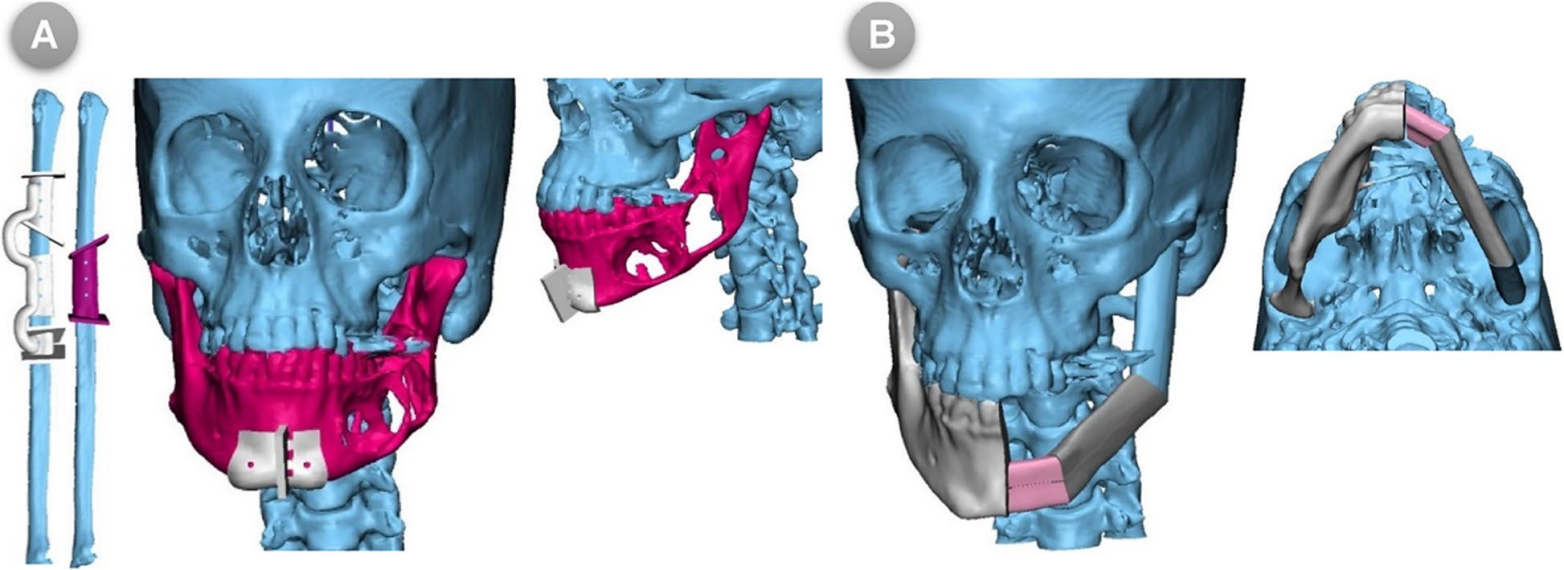
(**A**) 3D designs of mandibular and fibular cutting guides, (**B**) Virtual reconstructed mandibular models

Virtual mandibulectomy was performed according to the pre-determined plan and mapped resection osteotomies that were planned in accordance with the principles of radical tumour resection and confirmed in the inter-team communication sessions **(**Fig. 1B**)**. Once the extent of the defect had been clearly defined, the virtual reconstruction phase had begun. For the study (COG) group, the 3D virtual models were clearly reviewed and used to map the segments’ size, number, and shape of fibular bone cuts. Next, the patient’s 3D-reconstructed fibula was precisely superimposed on the mandibular defect. Fibular osteotomies were then virtually validated to recreate and renovate the native mandibular contour through a trial-and-error process, which was accomplished if the outer contour of the mandible was preserved. Otherwise, if destroyed by the tumor, a mirroring based-image was used to form the ideal mandibular contour by replicating the corresponding contralateral anatomy **(**Fig. 1C & D**)**. After that, a 3D-model of the virtually reconstructed mandible with fibular bone was created for the COG group. While, for the MB group, just a mirroring-based 3D-model of the mandible was created with the resection margins marked over the model. The virtually approved resection/reconstruction data were accordingly used to design and fabricate surgical cutting/osteotomy guides for both planned tumour resection and fibular osteotomies **(**Fig. 2A, B**)**.

#### Surgical Phase and post-operative data collection

**Fig. 3:**
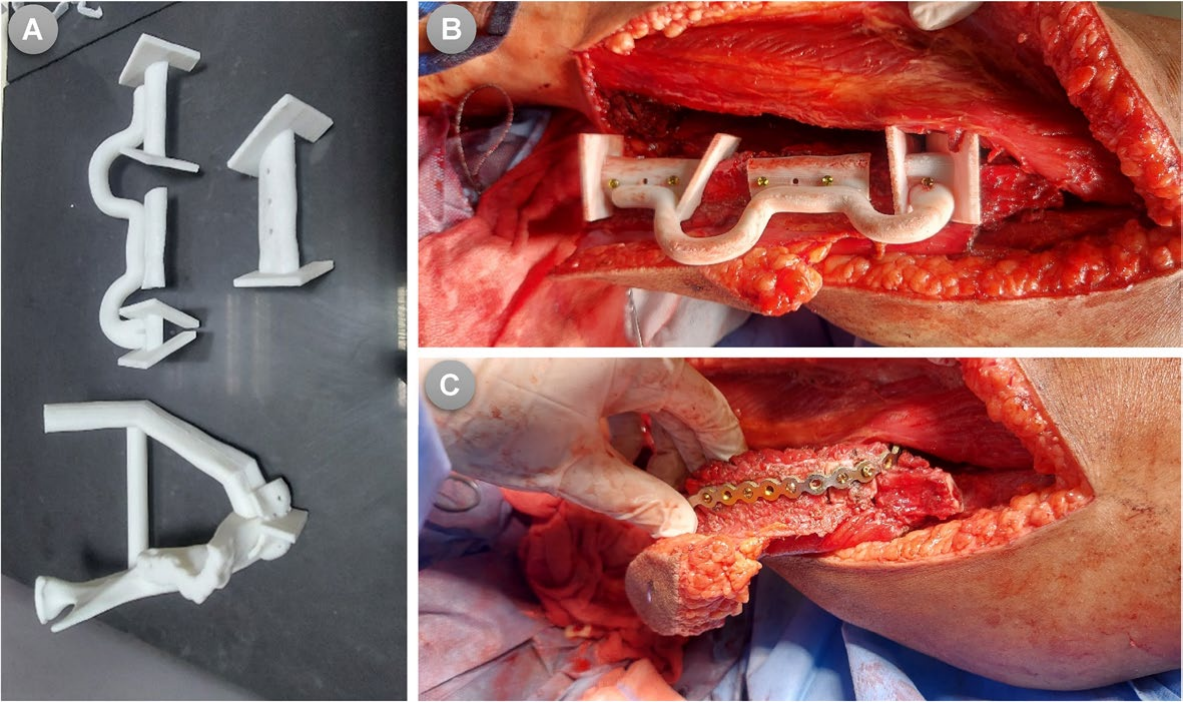
(**A**) Cutting guides and Model of the reconstructed mandible for another case, (**B**) Cutting guide was fixed on the fibula, (**C**) Osteotomies performed and fibular segments assembled into the neomandible before clamping the pedicle

The surgical procedures were performed by two surgical teams working simultaneously, one in charge of cervicofacial resection and the other for harvesting the flap. The extent of surgery and thus the approach to access the resection block were determined based on each patient’s case specifications. After neck dissection (where indicated) and access to the mandible were obtained, the surgical field was exposed and the tumour was surgically approached and explored. The customized 3D mandibular guiding templates for tumor resection were then applied and secured by monocortical screws in the predesignated virtually planned position. The osteotomies to resect the tumor were adopted either using a surgical saw or by Lindemann bur. Following the complete tumor resection and ensuring that radicality obtained, the recipient’s surgical bed was clear and ready for reconstruction procedures. The vascularized FFF was concurrently prepared by the second surgical team in charge of flap harvesting. In all patients, the FFF harvests were performed using the lateral approach. The procedure for cutting, conformation, and fixation of the flap varies between the two groups. In the COG group, the fibula was fully prepared and contoured at the donor site prior to vascular pedicle clamping according to the pre-operative planning using the COG. Before the execution of fibular osteotomies, the osteotomy guiding templates were temporarily adapted on the fibula to validate and confirm its accurate position. Once the correct position and orientation of the guide were affirmed, it was then secured accurately to the fibula using monocortical screws. In-situ, fibular osteotomies were then executed using a Lindemann bur inserted through the cutting slots where deperiostation had been performed. On completion of the guided fibular osteotomies, the cutting guides were removed along with the fixation screws from the fibula. Proceeding in situ, the fibular segments were then aligned/reassembled together and secured to the pre-bent reconstruction plate that was moulded on the 3D prefabricated mandibular model. Thereafter, the neo-mandible construct was confirmed and kept ready for microvascular transfer to the defect **(**Fig. 3**)**. Once the recipient site had been ready, the vascular pedicle was detached.

**Fig. 4:**
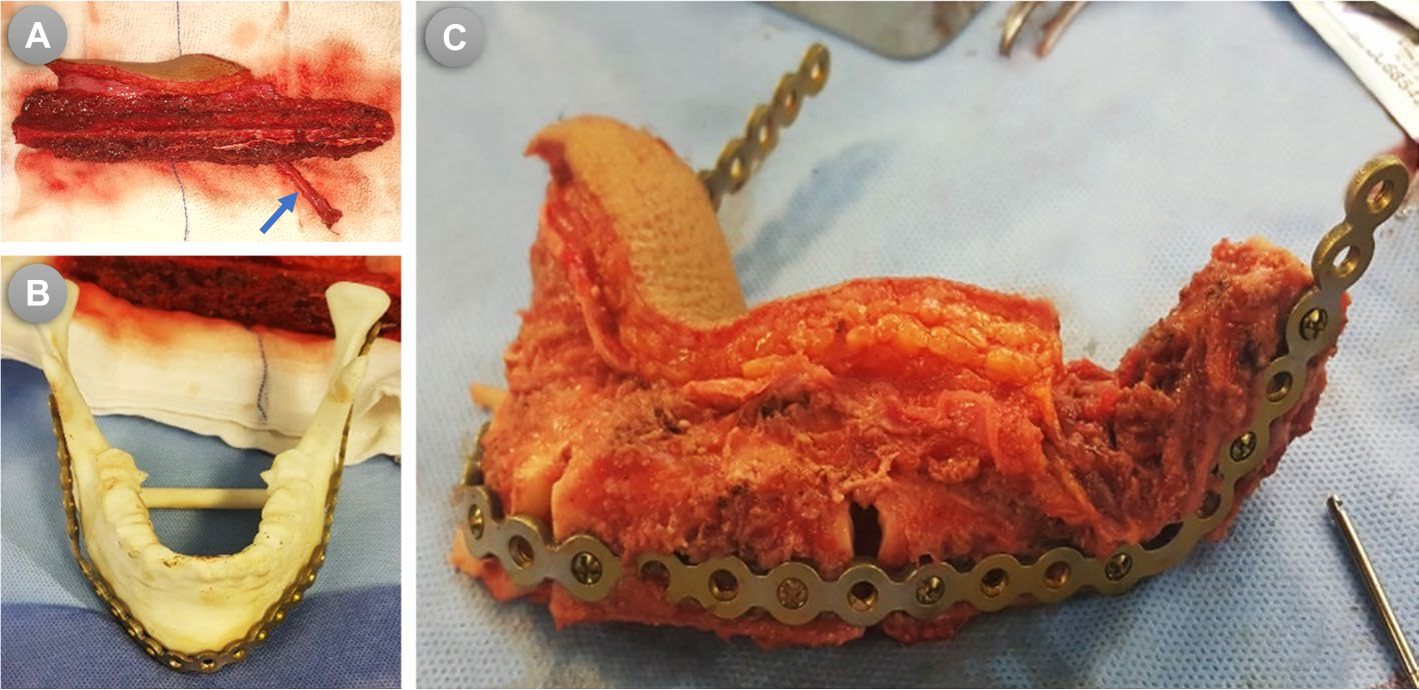
(**A**) Harvested fibula; blue arrow showed the FFF Pedicle detached, (**B**) 3D mandibular model, and (**C**) The fibular construct prepared on the back table

In the MB group, the same procedures for surgical resection and reconstruction were adopted, except that no customized cutting/osteotomy guides have been used neither for the mandibular resection nor for the fibula osteotomies. Only a 3D guiding template (model) for the mandible was virtually designed by mirroring the contralateral intact side and acquired to assist in plate conformation and accurate positioning. In this group, the fibula was fully prepared and contoured (construct) after the vascular pedicle had been detached. Once the FFF had been harvested, it was brought to the back table to perform the osteotomies without using osteotomy guides and depending on the surgeon’s experience and skills guided by the 3D-model of the mandible to confirm adequate contouring **(**Fig. 4**)**.

The flap construct was then transferred to the defect area, and reconstruction was achieved by securing the osseous construct with the plate to the native mandibular segments at its virtually pre-planned ideal position. Thereafter, the microvascular anastomoses were established. All patients in both groups received conventional free flap post-operative care and medications. During the routine follow-up visit in the 3^rd^ month after the operation, a CT scan was performed to obtain the post-operative data for evaluation.

#### Outcomes

The primary endpoint to be assessed was aesthetic outcome within 3 months of surgery. The post-operative aesthetic evaluation was performed objectively by the acquisition of a high-resolution craniofacial CT-scan to be used in outcome estimation by CDIA. The resultant measurements for each parameter were then rigorously calculated, summarized, and compared as a differential area (DAr) in square millimeters (sq. mm/mm^2^) and as differential angles (DAn) in degrees.

#### Differential area (DAr) measurements

**Fig. 5:**
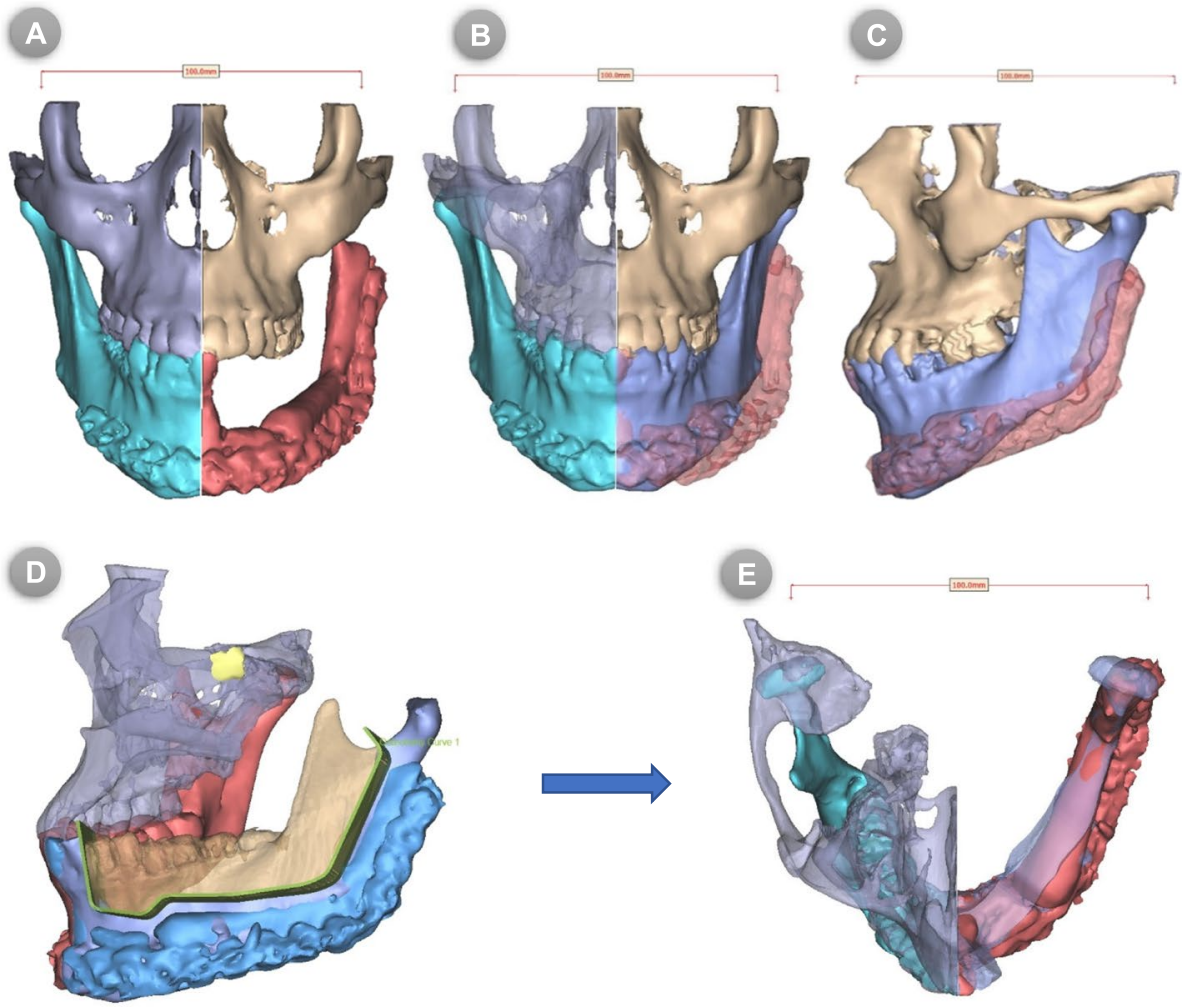
(**A**) Post-operative 3D virtual models of the maxillofacial skeleton, (**B**) The contralateral side of the mandible was simply mirrored and superimposed on affected side with transparency function applied, (**C**) Lateral view, (**D**) Virtual osteotomy in axial plane to reveal axial DAr, (**E**) Axial view

The post-operative CT-scan images in the DICOM format were imported into Synthes ProPlan CMF™ software and processed for the production of 3D virtual models of the craniofacial skeleton. The contralateral native side of the mandible was simply mirrored and superimposed on the affected side with the transparency function applied. A digital (virtual) reference in form of a measured bar was then placed and endorsed for real-size transfer and calibration during DAr measurements in sq. mm. In the axial view, a virtual axial cut was made in the contralateral mandible that was superimposed on the reconstructed side in order to clearly identify and measure the contour differences at the level of the fibula **(**Fig. 5**A- E)**. The resultant mirrored-based superimpositions in both sagittal and axial planes, including the endorsed digital reference, were captured and saved as two-dimensional (2D) images.

**Fig. 6:**
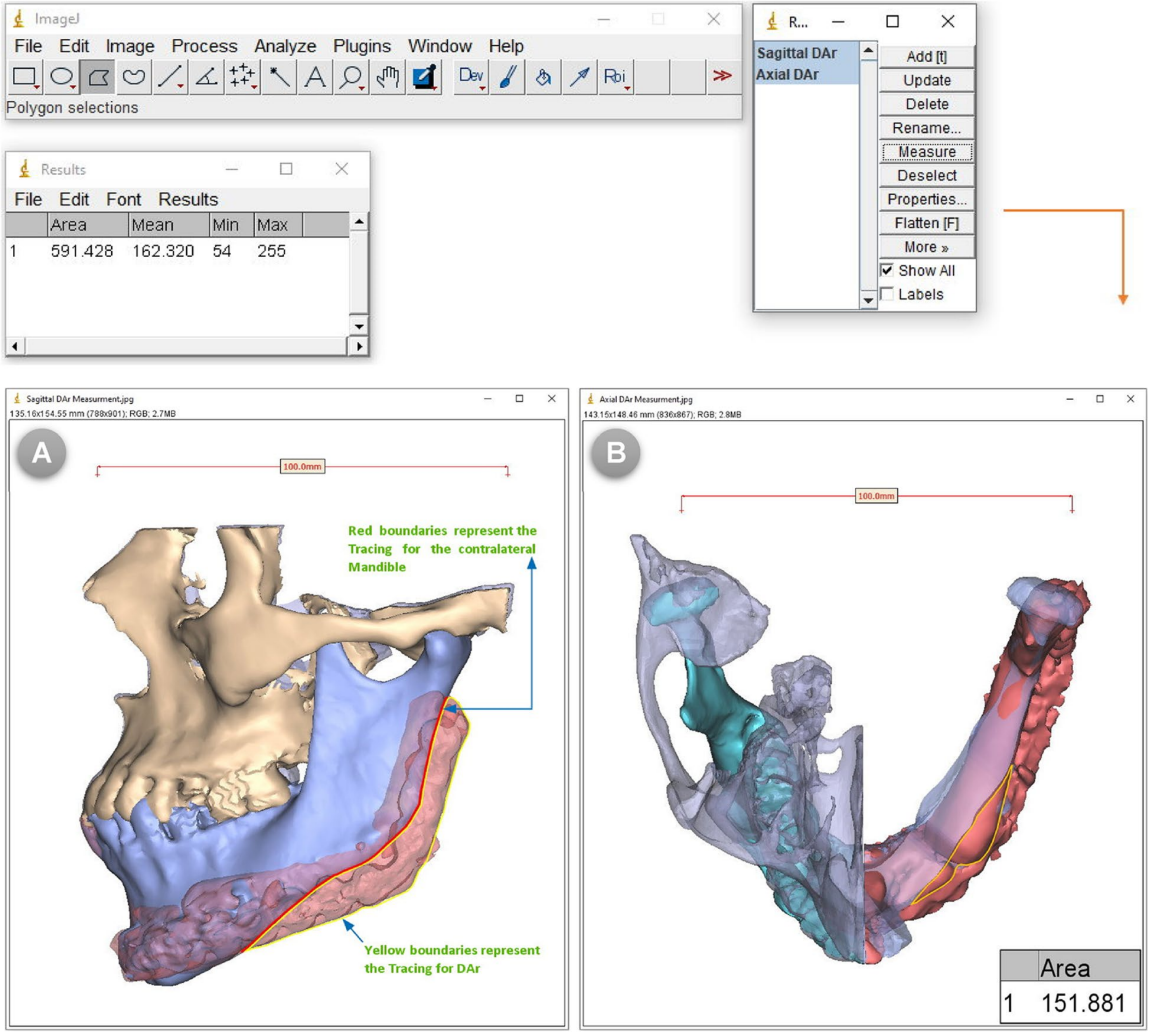
Differential area measurements performed using ImageJ software in both (**A**) Sagittal, and (**B**) Axial view

The obtained 2D-images were imported directly to ImageJ software V. 1.53 k (Wayne Rasband and contributors, National Institutes of Health, USA). The DAr was then traced and delineated using the Polygon selection tool and analyzed using the measure function tool after calibration by set scale function based on the digital reference placed previously; this was easily accomplished when modulated in ROI Manager **(**Fig. 6A, B**)**.

#### Differential Angle (DAn) measurements

**Fig. 7:**
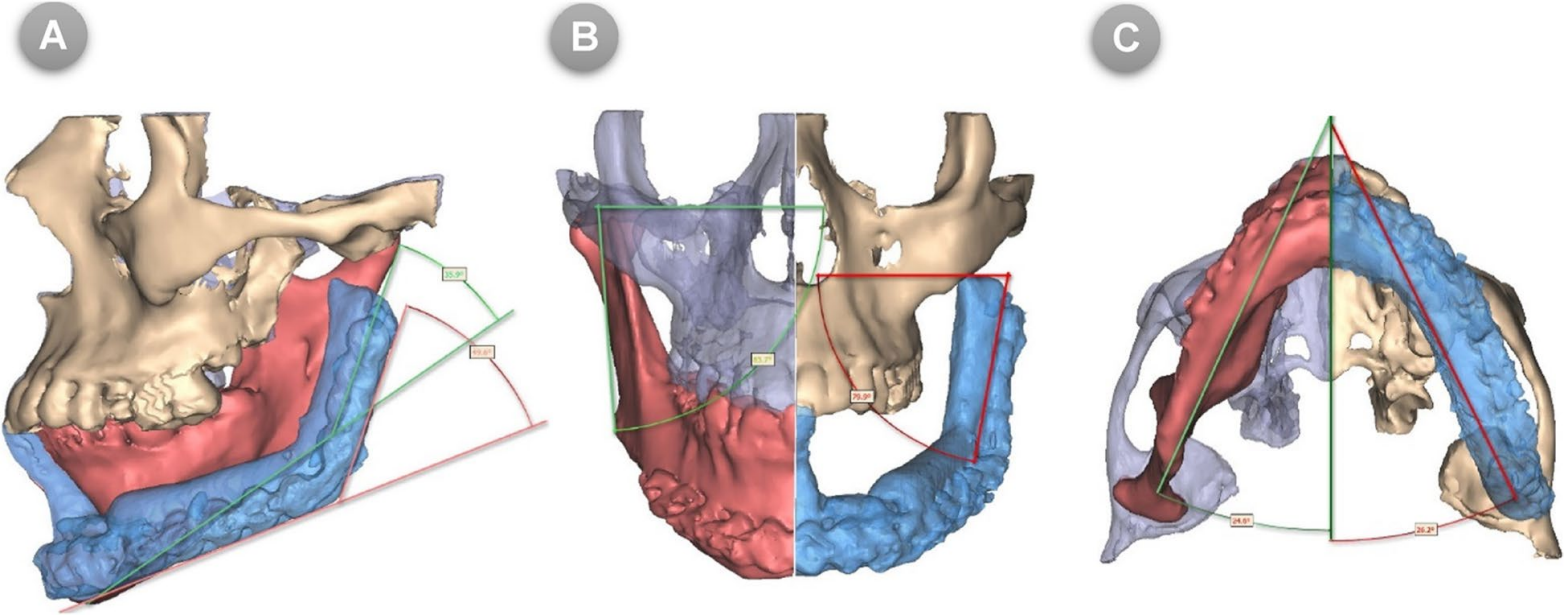
Differential Angles measurements performed using ProPlan CMF software for (**A**) Sagittal, (**B**) Coronal, and (**C**) Axial angles. NOTE: Sagittal angle is formed by the plane passing through the gonion (Go) and parasymphysis (ParaSym) and the plane passing through the Go and condylion (Co); Coronal angle is formed by the line passing through the two condyles parallel to Frankfort horizontal plane and the ascending ramus; Axial angle is formed by the plane passing through the Go and ParaSym and the midsagittal plane

Angular measurements based on the 3D-CT imaging were used to evaluate the morphologic restoration and degrees of asymmetry after reconstruction. Morphometric comparisons were made between the affected and the contralateral side of the reconstructed mandible by calculating differences in angular parameters **(**Fig. 7**A- C)**. Results were recorded as DAn (mean difference) in degrees, which are important for the facial profile. The greater the difference, the greater the surgical impact on mandible morphology. Symmetry was also assessed by calculating the ratio of the reconstructed to the contralateral side for the different angles. The closer the ratio to 1, the better the symmetry or the less the surgical impact on symmetry.

#### Subjective evaluation

All patients were independently assessed and screened clinically by two evaluators (the oncologic surgeon and maxillofacial professional). The esthetic outcome seen in the patients was then scored and indicated as a grade for each patient using a Visual Analogue Scale (VAS), which ranges from 0 to 10, where one near 0 represented a poor outcome and one near 10 represented an excellent esthetic outcome.

Subjective aesthetic evaluation has been carried out also using patients’ perceptions of the aesthetic outcome measured as Patient’s satisfaction Score (PSS), in which the patients had expressed their attitude and degree of satisfaction or dissatisfaction**.** In order to assess the patient’s satisfaction, a standard size mirror (size A5) was given to the subjects for assessment. Then the patients were instructed to score or put a mark on the scale that best reflected their satisfaction with the aesthetic result. The scored results were expressed in numbers from zero (worst aesthetic) to 10 (best aesthetic).

#### Operation time evaluation (secondary outcome)

Regarding the surgical efficiency, total operation time and ischemia time were assessed as secondary endpoints. Total operation time was the duration from the moment of surgical incision to the end of wound closure, which included the time for tumor resection, flap harvesting, and reconstruction. Since our study included both benign and malignant tumor cases, the neck dissection time was not included in the total operative time. The ischemic time was the time between the flap’s pedicle detachment and re-anastomosis. The entire surgical procedure was timed and recorded as a final value on a timing sheet in minutes (min) and summarized as a mean.

### Statistical methods

Based on a previous study by Azuma et al. in 2014 [13], the clinical important difference in DAn between the two groups was expected to be 6, with a standard deviation (SD) of the control group was ±4.38. Considering a significance level of 5.0% and statistical power of 80.0%, 9 patients per group would be necessary (18 totals). Taking into account possible losses to follow-up, this number was increased to a sample size of 11 patients per group (22 totals; 20% more than the calculated) for losses compensation. The sample size was calculated using PS program.

Data analysis was conducted using IBM SPSS advanced statistics, version 24 (SPSS Inc., Chicago, IL). Summary statistics were presented with either the mean ± standard deviation or the median (range) for numerical data and with counts and percentages for categorical data. Kolmogorov-Smirnov test and Shapiro-Wilk test were used to explore the data for normality. The Student’s t-test was used to compare the normally distributed numeric variables of aesthetic outcomes and operation times, while comparisons for non-normally distributed numerical variables were done by Mann-Whitney test. Comparisons between categorical variables were performed using the chi-square test. A *P*-value less than or equal to 0.05 (*P* ≤ 0.05) was considered statistically significant. All tests were two-tailed.

## Results

### Patients’ clinicodemographic data

**Fig. 8:**
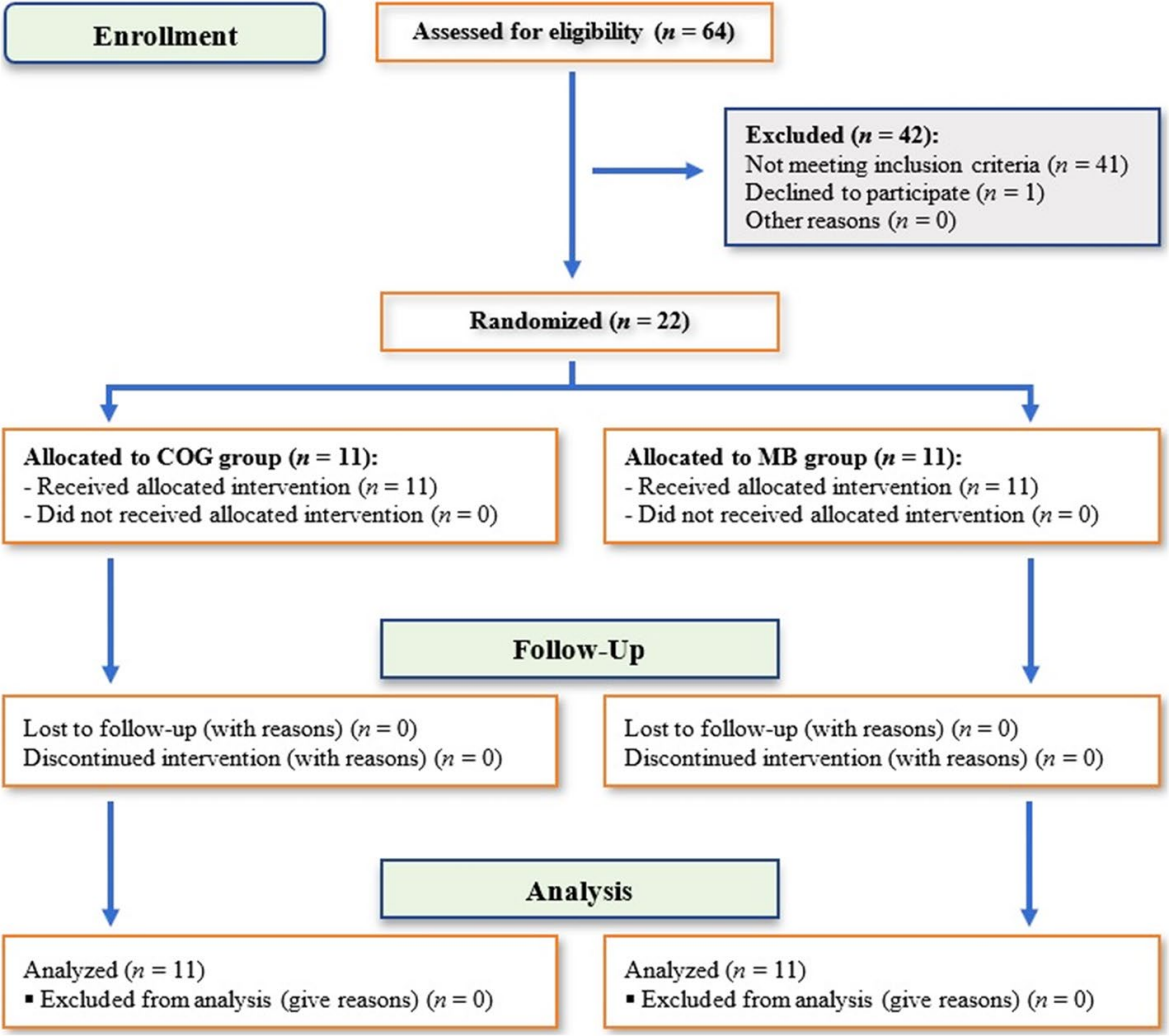
CONSORT flow diagram showing phases of the RCT

Of 64 patients screened for eligibility, 42 were ineligible. Twenty-two patients were randomized in equal numbers to the two study groups (*n* = 11 patients/group). Fig. 8 shows the consort flow chart of the study. Comparison of the two groups yielded the following results Table 1. The mean age in the COG group was 41 ± 18.5 years (range, 10- 63 years) versus 47.81 ± 13.6 years (range, 29- 63 years) in the MB group.

**Table 1:** Clinicodemographic characteristics of both groups of patients enrolled in this study

Variable			COG group (N = 11)		MB group (N = 11)		***p-***value
			Count	%	Count	%	
**Gender**		**Male**	7	63.60	6	54.50	
		**Female**	4	36.40	5	45.50	
**Age (yr) (Mean ± SD)**			41	±18.54	47.81	±13.75	.339
**Indication / diagnosis**		**Benign**	3	27.30	3	27.30	
		**Malignant**	8	72.70	8	72.70	
**Site**		**L**_**t**_ **Mandible**	7	63.60	6	54.50	
		**R**_**t**_ **Mandible**	2	18.20	3	27.30	
		**B**_**i**_ **Mandible**	2	18.20	2	18.20	
**Mandibular Defect**	**Length**_**(cm)**_ **(Mean ± SD)**		8.50	±2.56	9.42	±4.65	.575
	**Classification**	**II**	5	45.40	5	45.40	
		**IIc**	2	18.20	1	09.10	
		**III**	3	27.30	2	18.20	
		**IV**	1	09.10	2	18.20	
		**IVc**	0	0.0	1	09.10	
**Flap harvest side**		**L**_**t**_ **leg**	6	54.50	7	63.60	
		**R**_**t**_ **leg**	5	45.50	4	36.40	

**Abbreviations:** *Bi* Bilateral; *CAD/CAM, CL* Classification, *COG* Customized osteotomy guide, *F* Female, *F* Fibula flap, *Lt* Left; *M* Male, *MB* Model-based, *N* Number, *Pt* Patient, *Rt* Right, *SD* Standard deviation, *Yr* Year

Based on Brown’s classification of mandibular defects, in the COG group, 5 (45.4%) patients had CL II, 2 (18.2%) patients had CL IIc, 3 (27.3%) patients had CL III, and 1 (9.1%) had CL IV. While in the MB group, 5 (45.4%) patients had CL II, 1 (9.1%) patient had CL IIc, 2 (18.2%) patients had CL III, 2 (18.2%) patients had CL IV, and 1 (9.1%) had CL IVc. The mean mandibular defects’ length was 8.5 ± 2.56 cm in the COG group versus 9.42 ± 4.65 cm in the MB group. Regarding indication for reconstruction, 6 patients (27.30%) had benign lesions; of which, 5 were ameloblastomas, and 1 was a non-ossifying fibroma. Malignant tumors account for (72.70%) of the patients. The most common diagnosis was squamous cell carcinoma accounting for (*n* = 11; 68.75%) of the malignant neoplasms, all were stage IVA for their respective malignancy. The remaining malignancies account for (*n* = 5; 31.25%) and include; low-grade osteosarcoma (stage IA; *n* = 2), Ewing sarcoma (*n* = 1), grade I fibrosarcoma (*n* = 1), and 1 case was diagnosed as low-grade central mucoepidermoid carcinoma. Concerning radiotherapy, 12 patients (6 per group; 75%) received postoperative irradiation according to an oncologic adjuvant therapy regimen. All participants in this study have adhered to and completed the follow-up visits and no one was lost to follow-up**.** Table 1 shows the clinicodemographic data and tumor characteristics of all patients.

### Aesthetic Outcomes

#### Differential Area (DAr)

Quantitively, the CDIA revealed that patients in the COG group showed improved aesthetic outcome (contour symmetry) regarding the sagittal DAr with a mean of (277.28 ± 127.05 mm^2^) compared to (398.67 ± 139.10 mm^2^) in the MB group. The difference in the sagittal DAr was statistically significant (*P* = 0.045). Although the axial DAr was better in the COG group than in the MB group, there was no statistically significant difference between the two groups (*P* = 0.206) Table 2.

**Table 2:** Comparison of differential area (Sagittal and Axial DAr, mean ± SD) according to group

Differential area (DAr)	COG group		MB group		***P-***value
	Mean (mm^**2**^)	± SD	Mean (mm^**2**^)	± SD	
**Sagittal DAr**	277.28	± 127.05	398.67	± 139.10	0.045*
**Axial DAr**	147.61	± 55.42	183.68	± 72.85	0.206

**Abbreviations:** *COG* Customized osteotomy guide, *DAr* Differential area, *MB* Model-based, *mm*^*2*^ Square millimeter SD Standard deviation; * Significant *P*-value

#### Differential Angle (DAn)

The mean difference (Δ) and thus deviation for the sagittal and coronal DAn were significantly lower in the COG group than in the MB group (6.11 ± 3.46 and 1.77 ± 1.12° vs 9.53 ± 4.17 and 3.44 ± 2.34°; *P* < 0.05), respectively. While in the axial DAn, there was no statistically significant difference between the two groups (*P* = 0.386). Although these results indicate that the mandibular contour symmetry was improved, and even that better symmetry results have been found regarding the sagittal, coronal, and axial mandibular angles in favor of the COG group. However, there were no statistically significant differences in the symmetry for sagittal, coronal, and axial angles between the two groups (*P* > 0.05) Table 3.

**Table 3:** Comparison of the mean difference (Sagittal, Coronal and Axial DAn) and symmetry according to group

Differential and symmetry angle _**(affected /contralateral side)**_		COG group		MB group		***P***-value
		Mean (°)	± SD	Mean (°)	± SD	
**Sagittal**	**DAn (Δ)**	6.11	± 3.46	9.53	± 4.17	0.049*
	**Symmetry angle**	1.09	± 0.13	1.15	± 0.19	0.389
**Coronal**	**DAn (Δ)**	1.77	± 1.12	3.44	± 2.34	0.046*
	**Symmetry angle**	1.00	± 0.03	1.03	± 0.05	0.083
**Axial**	**DAn (Δ)**	2.60	± 0.74	2.93	± 0.97	0.386
	**Symmetry angle**	1.05	± 0.10	1.11	± 0.09	0.185

**Abbreviations:** *COG* Customized osteotomy guide, *DAn* Differential angle, *MB* Model-based, Δ Mean difference; (°) Degrees; * Significant *P*-value

#### Subjective evaluation of aesthetic outcome by VAS and PSS

The mean assessment score of the aesthetic outcome in VAS was higher in the COG group than in the MB group (8.18 ± 0.75 vs 7.64 ± 0.84), respectively. However, there were no statistically significant differences between the two groups (*P* = 0.12). On the other hand, the mean PSS was better in the COG group than in the MB group (8.14 ± 0.67 vs 7.45 ± 0.79), with statistically significant differences reflecting enhanced aesthetic outcome and better satisfaction (*P* = 0.041) Table 4.

**Table 4:** Comparison of Visual analogue scale (VAS) and Patient’s satisfaction score (PSS) according to group

Subjective evaluation	COG group		MB group		***P-***value
	Mean _**(score 1- 10)**_	± SD	Mean _**(score 1- 10)**_	± SD	
**VAS**	8.18	± 0.75	7.64	± 0.84	0.124
**PSS**	8.14	± 0.67	7.45	± 0.79	0.041*

**Abbreviations:** *COG* Customized osteotomy guide, *MB* Model-based, *PSS* Patient satisfaction score, *VAS* Visual analogue scale

**Fig. 9:**
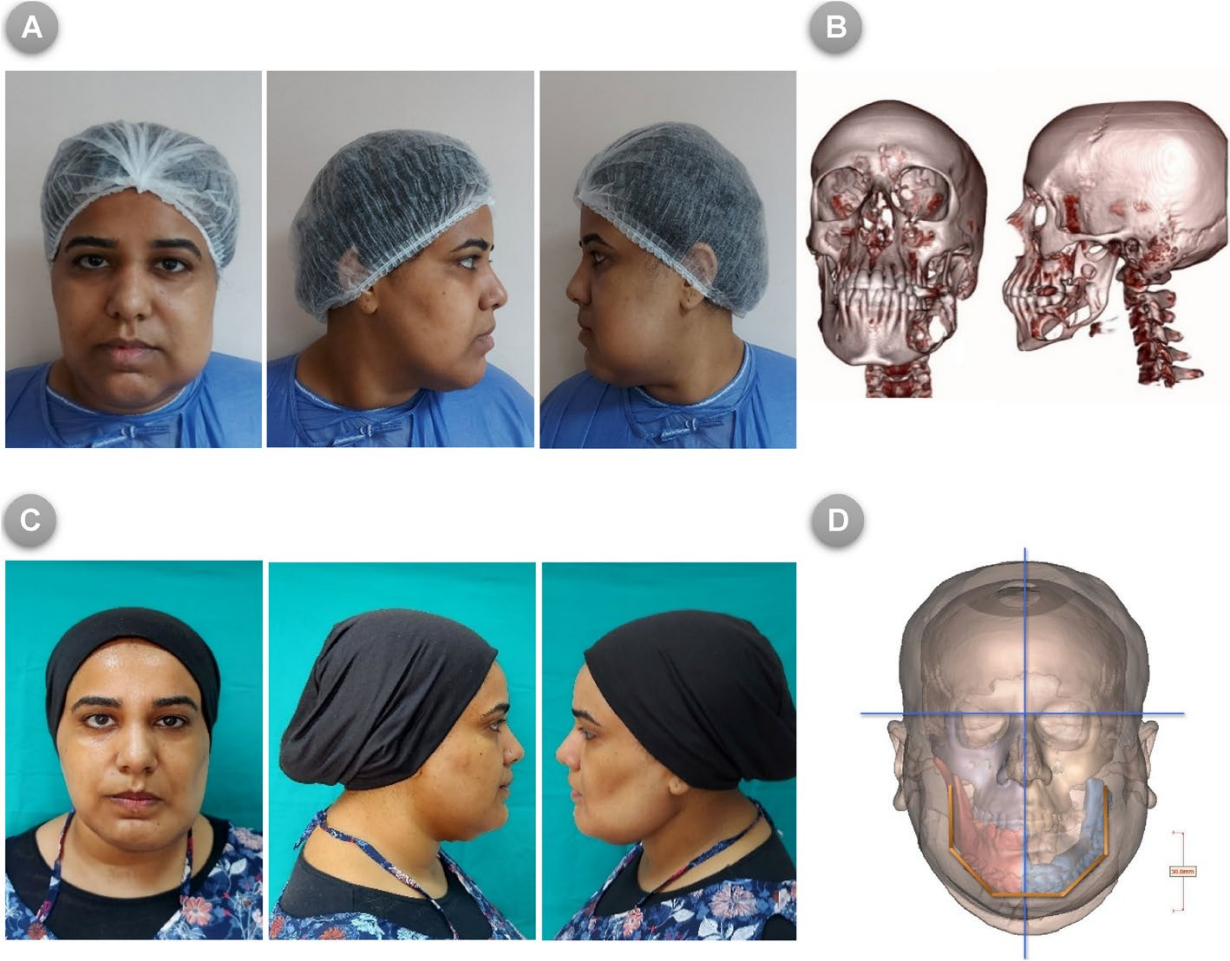
(**A**) Pre-operative photos for patient enrolled in the COG group (frontal and lateral views), (**B**) Pre-operative 3D CT scan image showing the defective mandible invaded by tumor, (**C**) Post-operative photos following reconstructive surgery using FFF with VSP and COG method, (**D**) Post-operative 3D CT scan demonstrating the newly reconstructed mandibular contour with soft tissue superimposition in frontal view

**Fig. 10:**
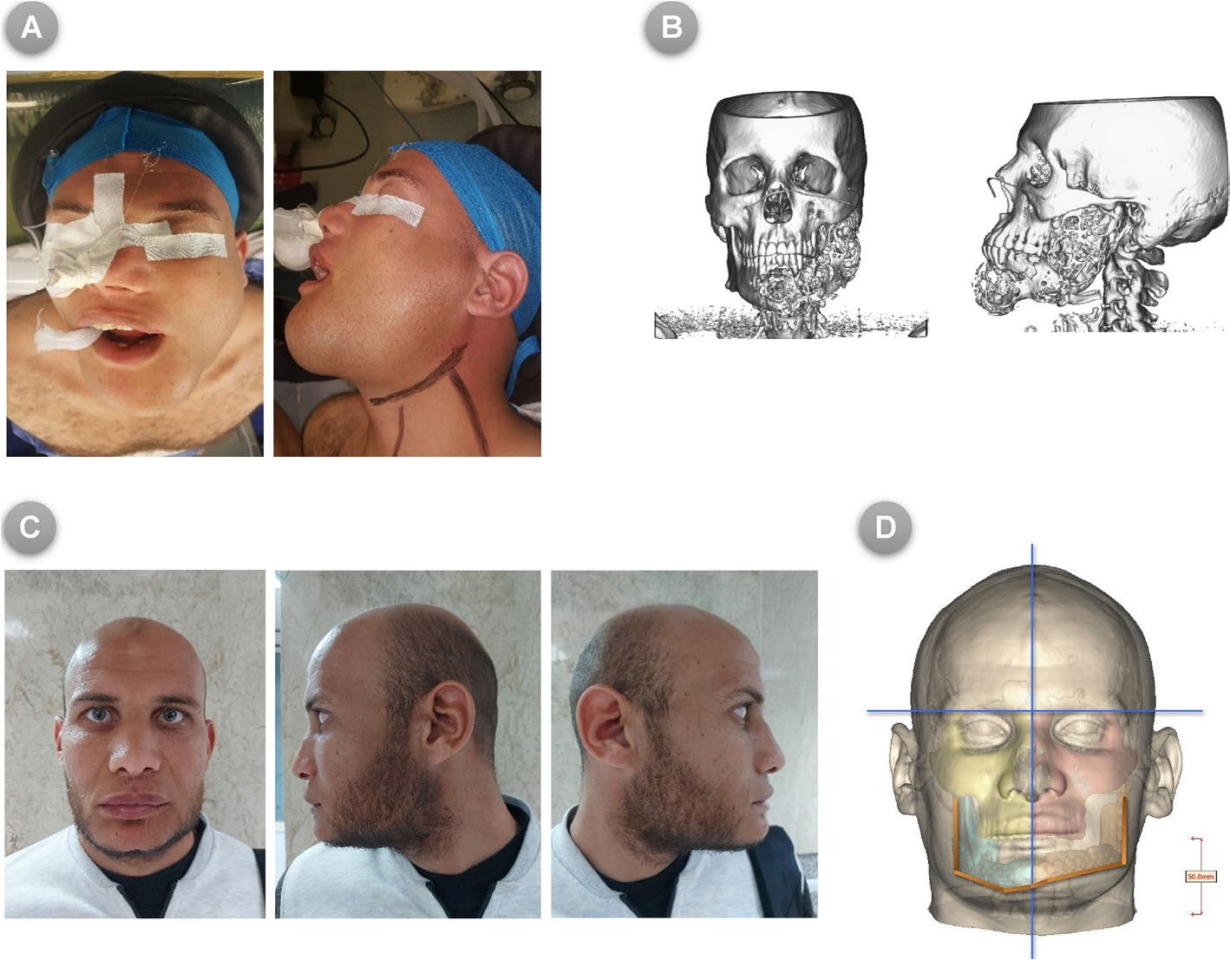
(**A**) Pre-operative photos for patient enrolled in the MB group (frontal and lateral views), (**B**) Pre-operative 3D CT scan image showing the defective mandible invaded by tumor, (**C**) Post-operative photos following reconstructive surgery using FFF with model-based reconstruction method, (**D**) Post-operative 3D CT scan demonstrating the newly reconstructed mandibular contour with soft tissue superimposition in frontal view

Representative patients receiving FFF reconstruction, either with the VSP and COG **(**Fig. 9**)** or Model-based technique **(**Fig. 10**),** are shown for mandibular contours and esthetics prior and after the surgical intervention.

#### Secondary outcome (operation and ischemic time)

In the COG group, the total operation time ranged from 467 to 645 minutes (562.91 ± 51.22 min, mean **±** SD) compared to 571- 728 minutes (663.55 **±** 53.43 min, mean **±** SD) in the MB group, there were statistically significant differences between the two groups (*P* = 0.0002). The mean ischemia time was 97.55 ± 16.80 minutes in the COG group, compared to 172.45 ± 21.87 minutes in the MB group. The difference in the ischemia time was statistically significant (*P* = 0.000) Table 5.

**Table 5:** Comparison of total operation time and ischemia time according to group (in minutes)

	COG group		MB group		***P-***value
	Mean _**(min)**_	± SD	Mean _**(min)**_	± SD	
**Total operation time**	562.91	± 51.22	663.55	± 53.43	0.0002*
**Ischemia time**	97.55	± 16.80	172.45	± 21.87	0.0000*

**Abbreviations:** *COG* Customized osteotomy guide, *MB* Model-based, *Min* Minutes

## Discussion

The mandible is an anatomically intricate structure, making ideal renovation and reconstruction extremely challenging. Suboptimal reconstruction may result in poor oral function as well as aesthetic deformities. Over the years, the microvascular fibular flap has been established as the workhorse for onco-mandibular reconstruction [5, 15]. However, the greatest challenge that remains is how to most accurately shape vascularized bone flaps so that facial symmetry, as well as function, are best restored and minimize the operative time of such complex surgeries [10]. Conventional techniques, either freehand or MB-approached, depend mainly on the surgeons’ experience and lack effective quantitative strategies [10, 16–19]. The advent of VSP, including CAD-CAM, has overcome the dilemma and changed the way of bony reconstruction in the past few years [5, 20, 21]. This randomized controlled trial aimed to evaluate and compare the aesthetic outcome and surgical efficiency of FFF with and without CAD/CAM COG for reconstruction of mandibular defects.

In the present study, DAr in both sagittal and axial planes has been introduced as a new criterion for the evaluation of aesthetic outcome. DAn (Sagittal, Coronal, and Axial) has been a part of objective evaluation as well. On the other hand, a subjective dual assessment of the aesthetic outcome has been performed using VAS and PSS. These intended-to analysis parameters are particularly important for facial aesthetics because the maintenance of the mandibular-arch diameter and angles’ amplitude are fundamentally crucial to fully imitate the native mandible and achieve facial symmetry [22].

The main focus of this study was on the construct replacing the defect and harmony with the remaining native mandibular segments. From this perspective, aesthetic evaluation has been considered for the same reconstructed native mandible. Since the contralateral side might also deviate or rotate after reconstruction if the plate was inadequately applied. Thus, it was better to be considered and involved in the final aesthetic evaluation postoperatively as the preoperative contralateral side doesn’t really express the final situation that actually exists after reconstruction. From this point of view, our study was particularly intended to evaluate aesthetics rather than accuracy and compare the reconstructed side with the contralateral side of the same reconstructed native mandible.

Our study shows a significant improvement in mandibular contour symmetry regarding the sagittal DAr and thus a better aesthetic outcome in the COG group compared to the MB reconstruction group (*P* = 0.045). Likewise, the mean difference between the affected and the contralateral side and thus deviation was significantly lower in terms of the sagittal and coronal mandibular angle (DAn) in the COG group compared to the MB group (*P* < 0.05), suggesting better overall symmetry and, notably, enhanced condyle sitting using cutting guides. These findings are all the more interesting for the current practice and imperatively adherent to the crucial goal of reconstruction.

Earlier forms of evaluation were based on pantomography to evaluate mandibular symmetry [5, 13, 14]. Although image standardization was performed according to the authors. However, concerns regarding under- or over-estimation are unfortunately still present, possibly because the radiograph is a uni-directional image. Current methods of evaluation are based on 3D-imaging analysis. Nevertheless, standardization regarding the evaluation process is still a concern. In this study, standardization of the evaluation process was addressed and seems to be mostly in line with the Jove-published protocol by van Baar et al. [23] in terms of imaging, machine and setting parameters, defects classification, using image-based 3D medical software, natural head position, and axis orientation. However, some steps were not exactly adherent to the Jove- published protocol, as our evaluation is based mainly on comparing the reconstructed mandible to the contralateral native mandible postoperatively.

Thus far, few data have been reported concerning the reconstruction quality in terms of restoration of the native morphology and preserving symmetry. Almost all studies have compared the reconstructed mandible to virtual planning rather than to the contralateral native mandible, which could overestimate the undoubted benefits of virtual planning [11]. In this study, morphological evaluation was achieved by comparing the superimposed virtual images of the reconstructed side to the contralateral native side of the mandible using 3D-CDIA on a post-operative CT-scan for the selected parameters. Given that symmetry and thus aesthetics is the imperative goal rather than accuracy, that has been vastly reported. Results, either in the form of a mean difference or symmetry ratio, can indicate and estimate the aesthetic outcome.

In the relevant literature, several studies have addressed the valuable use of VSP for mandibular reconstruction. Weitz et al. [5] found significantly smaller differences between the pre- and post-operative angle of the mandible in the virtual group compared to conventionally treated cases, 4.5° versus 11.5°, which is a measure that strongly influences the aesthetic outcomes and consistent proportions of the lower third of the face. Similar to Jacek and Azuma [13, 14]**,** Weitz et al. used pantomographic analysis, and hence only used one angle to compare results. Zhang et al. [24] when studying outcomes between computer-aided group vs freehand reconstruction, compared the VSP to the post-operative mandible alone. Even though their results show improved angle deviation in favor of the CAD group, the impact on aesthetic estimation may be limited since post-operative comparison with the contralateral native mandible was not done. Ren et al. [10] mentioned that the mean differences between the pre-operative and post-operative gonial angles were significantly smaller in the computer-assisted group compared with the conventional group (*P* = 0.007).

Similarly, Yu et al. [25] found that the variation between the reconstructed and contralateral mandibular angles was significantly different, favoring CAD-based over the conventional group (*P* = 0.001). Similar to the present study, Bartier et al. [11] in a retrospective study provided interesting results in favor of cutting guides. The mean difference between pre- and post-operative values of the coronal mandibular angles was significantly lower in the virtual planning group than in the traditional freehand group. Likewise, he found equivalent results in both techniques regarding post-operative symmetry for the coronal and axial mandibular angles but was significantly better regarding the sagittal angle in the 3D group. In general, the results of the present study are mostly in line with those of the Bartier study. Variations could be attributed to that conventional technique was used as a comparative control group in the Bartier study versus a 3D MB group in the present study, which could minimize the difference. However, it is difficult to directly compare the results of the present study with the aforementioned studies as all were compared to conventional reconstruction as a control, and most were performed retrospectively with the variations in the methodologies and parameters included in the evaluation, and thus potentially underrepresents the actual relevancy to the current imperative goals. Additionally, there is either a lack or an unclarified method for standardization in most of the studies.

De Maesschalck et al. [26] found equivalent results in both techniques regarding the mean difference and post-operative mandible symmetry in terms of sagittal and axial angles between the groups. Similarly, Stirling Craig et al. [27] found similar results in both techniques regarding post-operative body-symphyseal angle on axial view and thus mandible symmetry. Generally, the findings of these studies are contradictory to the present study and other reports regarding the efficiency of VSP, particularly cutting guides in improving aesthetic outcomes.

During the measurement of Dar and Dan in the MB group, the bony contact gaps or spaces were already a part of the healing process and involved in the total measurement, but was not calculated as a separate entity. Some studies have evaluated intersegment space or gaps as an independent measurement [27], which may not correspond to real aesthetics and have less impact on the outcome than the differential angles and areas used in the present study. Our study was designed differently to measure the difference as a total, including the bony gaps or spaces. In Fig. 4c, these gaps at the time of the evaluation have been shown as being a part of the healing process (bridged by bone), and hence no gaps to calculate as a separate entity.

In terms of subjective aesthetic assessment (SAA), findings of the present study have shown comparable results in both groups regarding VAS scores (8.18 versus 7.64), respectively. However, the PSS was significantly better scored (8.14) in the COG group compared to (7.45) in the MB group. In contrast to the present study, Bouchet et al. [28] reported that aesthetic satisfaction by PSS was higher in the conventional group (a score of ≥7 was reported by 85% (11/13) patients in the conventional group vs. 58% (7/12) patients in the CAD/CAM group. Given that satisfaction with the aesthetic result is vastly subjective and strictly related to patients’ expectations, the results can surprisingly vary.

Regarding surgical efficiency, Chang et al. [29] found that VSP significantly decreased operation and ischemia times compared to the MB group. Much the same findings have been presented by Toto et al., in 2015 [30]. This was also observed in several other studies that compared VSP with conventional technique [1, 10, 24, 31, 32]. The present study showed that the mean total operation time and ischemia time were significantly shorter in the COG group compared to the MB group, which is consistent with those studies. In contrast to the present study, Yu et al. [25] and Bartier et al. [11], even though they used conventional technique as a control, they found that the mean operative time did not significantly differ between the groups, which is contradictory to our findings. This could be because of many parameters either directly related or unrelated to the use of cutting guides and their impact on surgery time. Although the surgical efficiency of VSP has been vastly investigated. However, our results have resolved the conflict that resurfaced in some reports.

Despite the functional outcome assessment was not part of this study, all patients included in the study did not complain of any difficulties with mouth opening or occlusion, nor were there any problems noted by the physician’s observation during the follow-up period. However, further assessment will be done after the dental rehabilitation.

To the best of our knowledge, this study is the first randomized controlled clinical trial (RCT) investigating the aesthetic outcomes of FFF for reconstruction of the onco-mandibular defects by using the VSP and cutting guides versus that of Model-based reconstruction.

## Conclusion

The results of the present study indicated that the CAD/CAM with COG enhanced the aesthetic outcome in patients undergoing mandibular reconstruction using FFF compared to that without COG (MB reconstruction). It also has been shown to significantly enhance the surgical efficiency by minimizing total operative time and ischemia time. The limitation of this study is the relatively small sample size and the need to use a second-party software for DAr evaluation. Our measurements considered underlying hard bony tissue; further studies are required to analyze their impact on overlying structures. Another potential limitation is the lack of long-term follow-up, further studies with long-term follow-up could be considered to validate aesthetic outcome.

## Data Availability

The datasets used and/or analyzed during the current study are available from the corresponding author on reasonable request.

## Abbreviations

CAD/CAM: Computer-aided design and Computer-aided manufacturing
CAS: Computer-assisted surgery
CDIA: Computerized Digital Imaging Analysis
COG: Customized osteotomy guide
CT: Computed tomography
DAr: Differential area
DAn: Differential angle
DICOM: Digital Imaging and Communications in Medicine
FFF: Free fibular flap
MB: Model based
NCI: National Cancer Institute
Min: Minutes
PSS: Patient’s Satisfaction Score
SAA: Subjective aesthetic assessment
SNOSE: Sequentially numbered, opaque sealed envelopes
Sq. mm/mm^2^: Square millimeters
VAS: Visual analogue scale
VSP: Virtual surgical planning
Vs: Versus
3D: Three-dimensional
Δ: Mean difference
°: Degree
